# MiR-181 Family Modulates Osteopontin in Glioblastoma Multiforme

**DOI:** 10.3390/cancers12123813

**Published:** 2020-12-17

**Authors:** Anantha Marisetty, Jun Wei, Ling-Yuan Kong, Martina Ott, Dexing Fang, Aria Sabbagh, Amy B. Heimberger

**Affiliations:** Department of Neurosurgery, The University of Texas MD Anderson Cancer Center, Houston, TX 77030, USA; amarisetty@mdanderson.org (A.M.); JunWei@mdanderson.org (J.W.); lkong@mdanderson.org (L.-Y.K.); mott@mdanderson.org (M.O.); Dfang@mdanderson.org (D.F.); Aria.Sabbagh@uth.tmc.edu (A.S.)

**Keywords:** microRNAs, miRNA, miR-181, osteopontin, macrophages, cancer stem cells, glioblastoma, glioblastoma-infiltrating macrophages (GIMs), osteopontin (OPN), reverse transcription polymerase chain reaction (RT-PCR)

## Abstract

**Simple Summary:**

MicroRNAs can silence a broad set of target genes that may benefit heterogeneous tumors like glioblastoma. We have previously shown that osteopontin has an oncogenic role and may have immune modulatory effects on macrophages. In the current study, we used miRNAs to target osteopontin in tumor cells and modulate immune cells to elicit an antitumor effect. Intravenous delivery of miR-181a to immune competent mice bearing intracranial glioblastoma demonstrated a 22% increase in median survival duration relative to that of control mice. The overexpression of miR-181a in tumor cells led to decreased OPN production and proliferation and increased apoptosis in vitro, and increased survival duration of the mice when compared to its controls. miR-181a controls osteopontin expression in tumor cells by regulating their proliferation and apoptosis.

**Abstract:**

MiRNAs can silence a wide range of genes, which may be an advantage for targeting heterogenous tumors like glioblastoma. Osteopontin (OPN) plays both an oncogenic role in a variety of cancers and can immune modulate macrophages. We conducted a genome wide profiling and bioinformatic analysis to identify miR-181a/b/c/d as potential miRNAs that target OPN. Luciferase assays confirmed the binding potential of miRNAs to OPN. Expression levels of miR-181a/b/c/d and OPN were evaluated by using quantitative real-time PCR and enzyme-linked immunosorbent assay in mouse and human glioblastomas and macrophages that showed these miRNAs were downregulated in Glioblastoma associated CD11b+ cells compared to their matched blood CD14b+ cells. miRNA mimicking and overexpression using lentiviruses showed that MiR-181a overexpression in glioblastoma cells led to decreased OPN production and proliferation and increased apoptosis in vitro. MiR-181a treatment of immune competent mice bearing intracranial glioblastoma demonstrated a 22% increase in median survival duration relative to that of control mice.

## 1. Introduction

One of the critical factors that inhibits the successful treatment of glioblastoma is a poor understanding of the tumor’s biology and associated microenvironment. Glioblastoma-infiltrating macrophages (GIMs) constitute the largest immune cell population within the tumor microenvironment [1,2,3] and are recruited to the tumor through specific signals [4]. Studies by our research group and others have shown that tumor cells reprogram these macrophages from an antitumor phenotype to a tumor-supportive phenotype [4,5,6]. Targeted ablation or modifications of the tumor-supportive program of the microglia and macrophages within in vivo mouse models reduce glioma invasion and enhance survival [7]. A microarray analysis from our laboratory showed that secreted osteopontin was the most significantly upregulated gene in GIMs originating from circulating monocytes relative to the precursor in the blood of matched patients; this was further validated by qPCR [8]. OPN has been shown to have an oncogenic role in a variety of cancers and may have immune modulatory effects on macrophages. OPN is a crucial chemokine for recruiting macrophages in glioblastomas and for mediating a cross talk between tumor cells and immune cells [9]. OPN acts through various integrin’s to mediate cellular processes like migration, adhesion, and survival [10]. Macrophages can constitute up to 20% of the immune population in gliomas and are recruited to the microenvironment by extracellular signaling molecules and cytokines [8]. Macrophages recruited into the tumor can be polarized to either a classical proinflammatory phenotype (M1) or the tumor supportive Phenotype (M2). The non-polarized macrophages are called M0. GIMs exhibit immune functions similar to non-polarized macrophages [8].

Current immune therapeutics are limited to cell surface-expressed targets. However, many desirable targets are present within cells, including immune cell populations. We previously targeted a variety of immune modulatory targets using miRNAs that can efficiently silence broad gene sets of interest that may benefit heterogeneous tumors such as glioblastoma [11,12,13]. In this study, our hypothesis was that miR-181 family members regulate OPN expression in both the context of direct glioma cells effects and the immune system and that the upregulation of miR-181 in tumor cells or the systemic delivery of the microRNAs will have anti-tumor effects. We tested this hypothesis in the immune cells by overexpressing miRNA mimics (miR-181a/b/c/d) in skewed macrophages and quantifying the expression levels of OPN in vitro and in glioma cells by establishing stably overexpressing miR-181a/b/c/d cell lines and quantifying the OPN expression in vitro. Thereafter, the miR-181a/b/c/d overexpression in glioma cells was assayed for changes in proliferation and apoptosis in vitro. The cells were then implanted in mice and survival was monitored. In order to determine if systemic delivery of miR-181 mimics has any antitumor effects in vivo we systemically delivered intravenously miR-181 mimics in C57BL/6J mice with established GL261 tumors.

## 2. Results

### 2.1. miR-181 Family Is Downregulated in CD11b+ Monocytes Extracted from Glioblastoma

**Figure 1:**
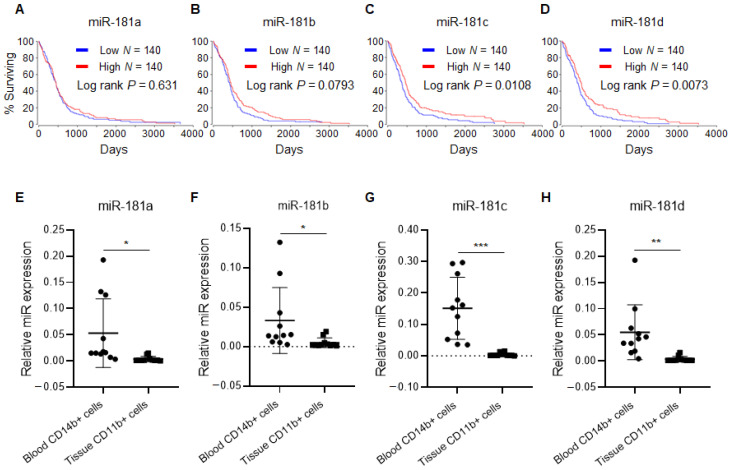
miR-181a/b/c/d expression are positive prognosticators in glioblastoma patients and is downregulated in CD11b+ macrophages from glioblastoma tumors. (**A**–**D**) miR-181a/b/c/d expression in glioblastoma tumor positively correlates with patient survival. Kaplan–Meier survival estimates of glioblastoma patients in relation to expression levels of miR-181a (**A**), miR-181b (**B**), miR-181c (**C**) and miR-181d (**D**) in the glioblastomas based on TCGA data sets. Low: miR-181a/b/c/d low-expressing (25%, *n* = 140) group; High: miR-181a/b/c/d high-expressing (25%, *n* = 140) group. Eleven glioblastoma specimens with matched CD14b+ cells from blood and CD11b+ cells from tumor specimens were profiled using quantitative PCR. (**E**) miR-181a, (**F**) miR-181b, (**G**) miR-181c, and (**H**) miR-181d expression levels were significantly downregulated in glioblastoma CD11b+ cells compared to in matched blood CD14b+ cells. Data is cumulative of 3 independent experiments run in triplicates. Statistical significance was determined using an unpaired Student’s t test. * *p* < 0.05, ** *p* < 0.01, *** *p* < 0.001. MiR-181 family regulates Osteopontin (OPN) in monocyte-derived macrophages.

TCGA data analysis show glioblastoma patients with higher miR-181 family expression in the tumor specimens survived longer (Figure 1) and this positive correlation is more significant for miR-181c and miR-181d (Figure 1C,D). Furthermore, a microarray analysis from our laboratory showed that OPN was the most significantly upregulated gene in GIMs that originated from circulating monocytes relative to the precursor in the blood of matched patients [8]. We validated this finding by determining the expression levels of OPN with qPCR in 11 glioblastoma patients and normal controls [9]. Utilizing predictive targeting programs, (TargetScan, miRDB, RNAhybrids, and miRbase) five miRNAs miR-4262, miR-181a, miR-181b, miR-181c, and miR-181d were predicted to bind to the 3′UTR of OPN (Table S1). A genome-wide miR expression analysis of GIMs relative to circulating monocytes demonstrated that the miR-181 family (a, b, c, and d) were all preferentially downregulated in these cells [12].

Given the redundancy of representation of the miR-181 family by target prediction programs and genome wide profiling data, we evaluated the expression of each miR-181 family member in matched blood and glioblastoma tissue in the CD14+ blood monocyte and CD11b+ tissue macrophage population. We showed that CD14 and CD11b stained the same macrophage population residing in the glioblastoma tissue [8]. In all instances, miR-181 family member expression was higher in matched blood than in glioblastoma (Figure 1E–H).

**Figure 2:**
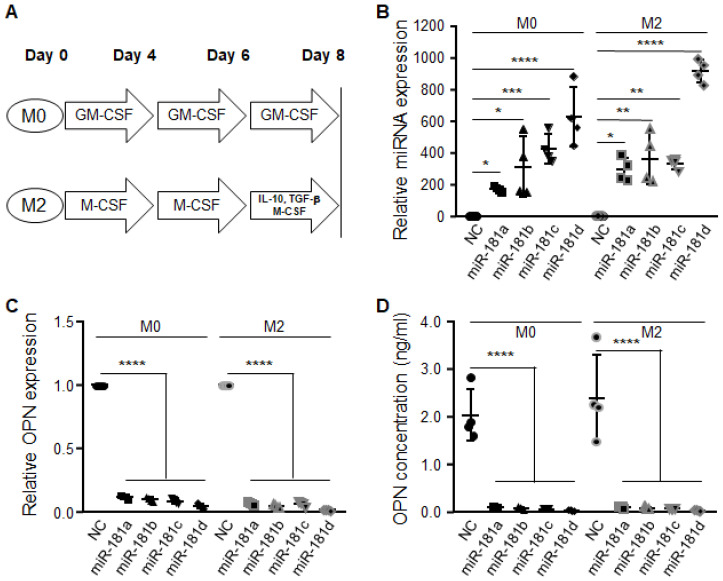
MiR-181 family regulates OPN. (**A**) Schema showing the derivation of M0 and M2 polarized macrophages that are the dominant phenotype of macrophages in gliomas. (**B**) Transient transfection of M0 or M2 macrophages on day 6 for 48 h with either non-targeting control (NC) or miR-181a/b/c/d-5p mimics. MiR overexpression levels were confirmed by qRT-PCR. (**C**) Analysis of the resulting cells revealed that overexpression of miR-181a/b/c/d mimics resulted in a decrease in OPN gene expression at the RNA level (qRT-PCR) and (**D**) the protein level (ELISA). Data were normalized against the levels of U6 small RNA for all qRT-PCR experiments. The data represent the mean ± SD (*n* = 3) in three independent experiments. Data is cumulative of 3 independent experiments run in triplicates. Statistical significance was determined using an unpaired Student’s *t* test. * *p* < 0.05, ** *p* < 0.01, *** *p* < 0.001, **** *p* < 0.0001.

We previously showed that macrophages skew heterogeneously but mostly exist in a continuum between M0 and M2 [8]. To determine the expression profile of miR-181 and OPN, we isolated normal donor peripheral blood mononuclear cells from the buffy coats of Ficoll gradients; the CD14+ cells were isolated; the macrophages were skewed to M0 and M2 and transiently transfected of miR-181a/b/c/d mimics (Figure 2A). In all instances, there was an upregulation of miR-181 expression in both the M0 and M2 cells (Figure 2B). When there was a forced overexpression of miR-181, OPN expression was eliminated, as detected by both quantitative PCR (Figure 2C) and ELISA (Figure 2D).

**Figure 3:**
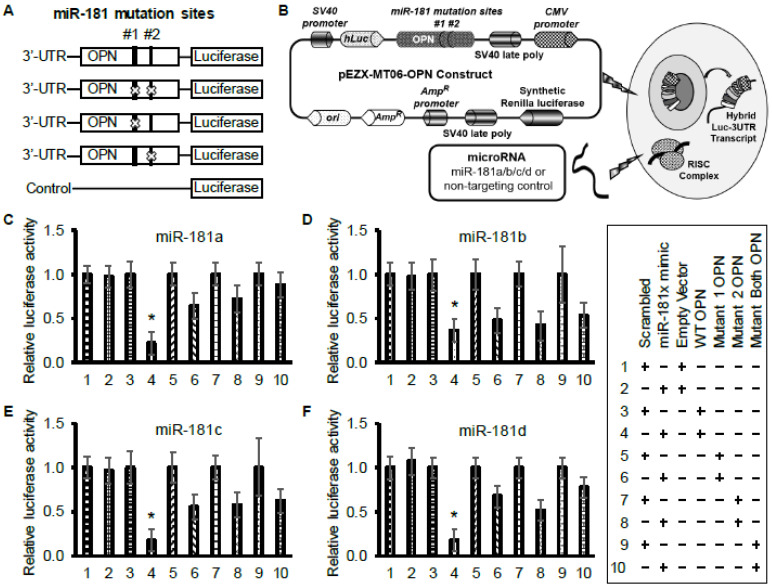
OPN is a direct target of miR-181a/b/c/d. (**A**) Schematic representation of miR-181a/b/c/d binding sites on the 3′-UTR of OPN and mutation of those sites by site-directed mutagenesis for the luciferase assay. (**B**) Schematic illustration of the vector backbone of miRNA-3′ UTR target clones and transfection into cells. (C–F) Dual luciferase reporter assay data showed that co-transfecting HeLa cells with WT-OPN 3′-UTR and with miR-181 family mimics resulted in a significant decrease in luciferase activity. Co-transfection with mutant 1, mutant 2, or dual-mutant-OPN3′-UTR with miR-181 family mimics resulted in no difference compared to the control group. The data represent the mean ± SE (*n* = 3) in three independent experiments. Statistical significance was determined using an unpaired Student’s *t* test. * *p* < 0.05.

Luciferase assays were performed to functionally validate the binding of the miR-181 family to OPN. As controls, we used reporter plasmids with no binding site or with a mutated binding site (Figure 3A,B). Cells were transfected with the reporter plasmids and microRNA mimics for each condition. Our results indicated that miR-181a-5p has a stronger binding capacity to OPN than the other miRs (Figure 3C–F).

### 2.2. MiR-181a Regulates the Survival of Immune-Competent Mice Harboring GL261 Brain Tumors

**Figure 4:**
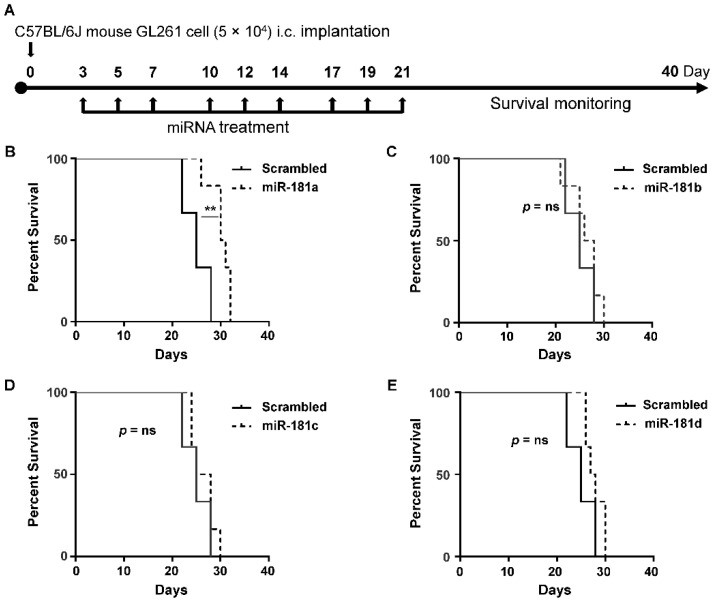
miR-181a regulates the survival of immune competent mice implanted with GL261 tumor cells. (**A**) Treatment schema for delivering the scrambled control or miR-181a/b/c/d-5p mimics intravenously in C57BL/6J mice implanted with intracranial GL261 gliomas. Kaplan–Meier survival analysis of mice harboring intracranial GL261 tumors treated with (**B**) miR-181a, (**C**) miR-181b, (**D**) miR-181c, and (**E**) miR-181d or scrambled control intravenously. Delivery of miR-181a-5p mimics improved survival compared to the scrambled control (*p* = 0.0062). Treatment with other miR-181 family mimics (miR-181b/c/d) did not improve survival. The Gehan–Breslow–Wilcoxon test was used to compare overall survival between groups (*n* = 6/group). Statistical significance was determined using an unpaired Student’s t test. ** *p* < 0.01.

We previously showed that when microRNAs are administered with lipofectamine, they preferentially target the monocyte populations; very little of the delivered microRNA is detected within gliomas [14]. To assess the in vivo efficacy of miR-181a/b/c/d-5p, we implanted the GL261 murine glioma cell line into immune-competent mice and treated them with either scrambled control or miR-181a/b/c/d-5p by intravenous injection three times a week for 3 weeks (Figure 4A). The mice treated with miR-181a-5p had a median survival duration of 30.5 days, while the control mice had a median survival duration of 25 days (*p* = 0.0062) (Figure 4B); treatment with other miRs had no statistically significant effect (Figure 4C–E).

**Figure 5:**
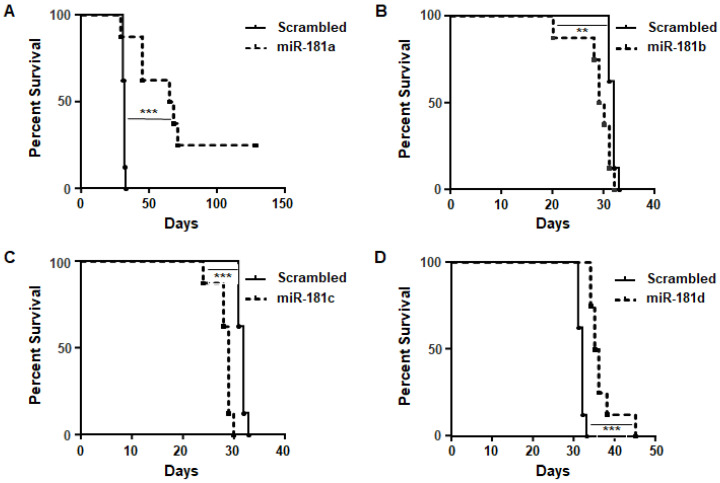
Overexpression of miR-181a regulates the survival of immune-competent mice implanted with miR-181a-overexpressing GL261 tumor cells. (**A**) miR-181a, (**B**) miR-181b, (**C**) miR-181c, and (**D**) miR-181e and scrambled miRNA controls (designated control) were overexpressed in GL261 tumor cells by lentiviral transduction. MiR-overexpressing cells were implanted intracranially in immune-competent mice, and mice were monitored for survival. Mice harboring GL261 miR-181a-overexpressing tumor cells survived for longer than control cells (median, 66.5 days vs. 32 days; *p* = 0.0017). The Gehan–Breslow–Wilcoxon test was used to compare overall survival between groups (*n* = 6/group Statistical significance was determined using an unpaired Student’s *t* test. ** *p* < 0.01, *** *p* < 0.001.

Since the miR-181 family had marginal therapeutic efficacy in immune-competent mice, we evaluated the direct effects of manipulating tumor cells with this miRNA family. Stable glioma cell lines overexpressing miR-181a/b/c/d were generated using lentiviruses for either the control or miR-181a/b/c/d. MiR overexpression (Supplementary Materials Figure S1A) and OPN knockdown (Figure S1B) were confirmed. The transfected cells were implanted in mice, and a Kaplan–Meier survival analysis was performed. The mice implanted with murine GL261 miR-181a cells survived for longer 66.5 days), while the control mice had a median survival duration of 32 days (*p* = 0.0017) (Figure 5A–D). Overexpression of miR-181b/c/d had a more modest impact on survival, indicating that miR-181a had additional modulatory effects beyond OPN (Figure S1).

### 2.3. Identification and Validation of miR-181a-5p Apoptotic Targets

**Figure 6:**
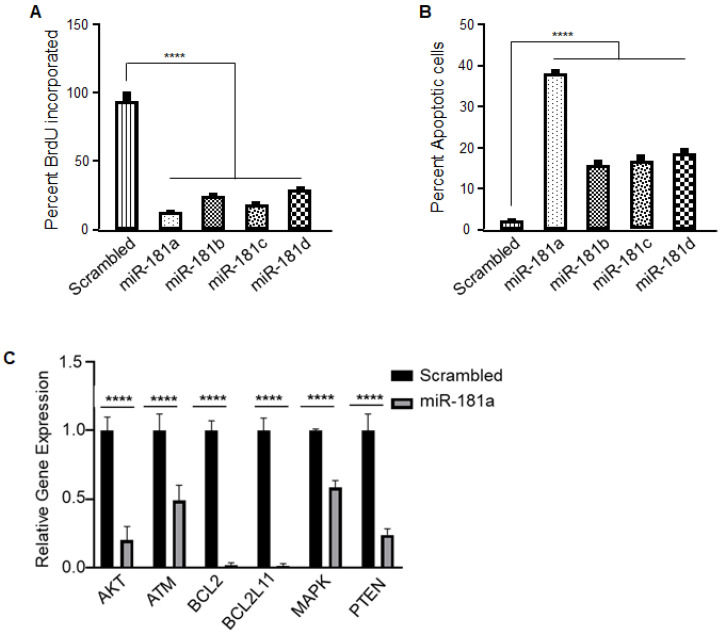
Identification of potential downstream targets of miR-181a-5p. (**A**) Cell proliferation was evaluated using a BrdU labeling assay. A decrease in proliferation was observed upon overexpression of the microRNA mimics, but a greater decrease was seen with Scrambled vs. miR-181a (*p* < 0.001), when compared to other miR-181 family members. miR-181a vs. miR-181b (*p* = 0.0213), miR-181a vs. miR-181c (*p* = 0.3662; ns), miR-181a vs. miR-181d (*p* = 0.0013). (**B**) Apoptosis in miR-overexpressing cells was measured using TUNEL assays. Overexpression of miR-181a significantly induced apoptosis in GL261 cells compared to other miRs (miR-181b/c/d) (Scrambled vs. miR-181a and miR-181a vs. miR-181b/c/d *p* < 0.001). (**C**) Transcripts were generated and analyzed for apoptotic targets (AKT, ATM, BCL2, BCL2L11, PTEN, and MAPK) by qRT-PCR from the miR-181a overexpression and scrambled control cells. The data represent the mean ± SE (*n* = 3) in three independent experiments done in triplicates Statistical significance was determined using an unpaired Student’s *t* test. **** *p* < 0.0001.

MiR-181a has been shown to promote apoptosis by increasing Fox01 acetylation [15] and by repressing *BCL-2* in cancer cells [16]. As such, we performed cell proliferation and apoptosis assays on miR-181a/b/c/d-overexpressing GL261 cell clones and observed that miR-181a led to a significant decrease in cell proliferation and an increase in apoptosis compared to other miR-181 family members (miR-181b/c/d) (Figure 6A,B), indicating that miR-181a has additional tumor suppressor functions in GL261 cells. This data is further supported by measuring the caspase-3 activity in the miR-181 overexpressing GL261 tumor cells (Figure S2). Upon miR-181a overexpression there is an increase in the caspase 3 activity when compared to its scrambled control (Figure S2).

To determine the potential apoptotic targets of only miR-181a-5p, we downloaded the predicted targets using miRDB (an online database for microRNA target prediction and functional studies: mirdb.org), which yielded 1120 genes. We identified 473 genes that are unique to miR-181a-5p but not to other miR-181 family members. Because the cells that overexpressed miR-181a-5p were undergoing apoptosis, the panel was focused on genes that induce apoptosis in cancer cells. The gene list was compared to a publicly available apoptosis gene list, which further narrowed it to 38 genes. Further, on the basis of a literature review, we determined that miR-181a-5p likely mediated the apoptosis in GL261 cells through multiple candidates, including BCL family members such as BCL2 and BCL2L11, PTEN, ATM kinase, and MAP kinases. These miR-targets were validated by generating transcripts from the miR-181a overexpressing cells and analyzing them by quantitative real time PCR. We first confirmed the expression levels of miR-181a and OPN (Figure S1) in these cells and then looked into potential apoptotic targets (Figure 6C). We analyzed the expression levels of these six miR-targets (BCL2, BCL2L11, PTEN, ATM, AKT, and MAP) based on the binding potential and literature and found that all of them were significantly downregulated upon miR-181a overexpression compared to its scrambled control (Figure 6C). Thus, these results support AKT, ATM, BCL2, BCL2L11, MAPK, and PTEN as downstream targets that mediate apoptosis in miR-181a overexpressing cells.

## 3. Discussion

In both glioma cells and macrophages, miR-181 family members (miR-181a/b/c/d-5p) target OPN expression. The miR-181 family has been previously shown to inhibit cancer stem cell functions [17] and invasion/metastasis [18,19]. miR-181 can also inhibit malignant transformation of gliomas by targeting KPNA4 [17,18,20]. Based on the results of predictive algorithm strategies, microarray data, and functional luciferase assays, members of the miR-181 family bind to OPN and regulate its expression. Furthermore, the overexpression of miR-181a-5p induces apoptosis and decreased proliferation in glioma cells. These findings would be consistent with other studies that have shown the miR-181 can inhibit glioma growth, proliferation, and invasion by targeting F-box protein 11 and CCL8 [21,22]. miR-181a-5p mediated apoptosis in GL261 cells through multiple candidates, including BCL family members such as BCL2 and BCL2L11, PTEN, ATM kinase, and MAP kinases. Bcl-2 has been identified as a potential target of miR-181a-5p [23,24,25]. Bcl-2 is a pro-survival molecule that is overexpressed in many cancers. It not only contributes to tumor initiation and progression but also to chemotherapy resistance [26]. In this study, we observed that the overexpression of miR-181a-5p results in an increase in apoptosis, which may be due to the downregulation of the potential target Bcl-2, an anti-apoptotic factor. Gl261 tumors have been reported to have higher expression levels of Bcl-2 [27]. Studies have also shown that the miR-181 family can inhibit cell proliferation and promote apoptosis by targeting PTEN [28] by targeting SELK in gliomas [29]. Conversely, miR-181a acts as an oncomir in gastric cancers. In this context, the overexpression of miR-181a promotes proliferation and inhibits apoptosis by directly targeting ATM [30].

Previously, we showed that OPN was upregulated in CD11b+ cells that were isolated from gliomas. In the present study, we show that the expression levels of the miR-181 family were decreased. In M0 and M2 macrophages, which are the predominant immune cell population in glioblastoma [8,31], overexpression of the miR-181 family decreases OPN expression, which drives glioma cell survival and stimulates angiogenesis [32]. In addition, OPN is involved in mediating chemoresistance [33]. Han et al. showed that miR-181c directly targets OPN and that its overexpression significantly inhibited breast cancer cell proliferation, reversed doxorubicin chemoresistance, and reduced breast cancer xenograft growth [34]. OPN is a pleotropic factor and can be produced by a variety of cell types under a range of physiological situations [35]. We showed that both tumor-derived and host-derived OPN are critical for glioblastoma development. The elimination of OPN expression in glioblastoma cells or non-tumor cells resulted in a marked reduction in the number of M2 macrophages and elevated T cell effector activity. Furthermore, we showed that glioblastoma-infiltrating macrophages are the major source of the host-derived OPN in the tumor microenvironment [9]. Thus, it is critical to target OPN in the glioblastoma microenvironment to further enhance the miR-181 family’s anti-glioma efficacy. This can be achieved by miRNA therapeutic enrichment in the brain tumor microenvironment via intracranial administration or tumor-specific cargo delivery strategies, such as aptamers and nanoparticles.

The systemic intravenous delivery or administration of miR-181a-5p, but not miR-181b/c/d-5p, in mice harboring GL261 tumors increased survival by 22%. Notably, the therapeutic effect of direct OPN genetic modification through shRNA was much more robust in the syngeneic murine model grafted with OPN shRNA GL261 cells [9]. Since a systemically administered miRNA strategy would not significantly influence OPN expression in glioma cells and the tumor microenvironment, we genetically modified GL261 tumor cells by stably overexpressing the miR-181 family members individually. We then implanted the cells in immunocompetent mice and monitored their survival. The median survival duration of mice with miR-181a-5p overexpression was doubled that of the scrambled control, while other miR-181 family members had a more modest survival impact, again emphasizing that miR-181a-5p has a different impact relative to that of other family members, regardless of the modulation site (i.e., tumor versus systemic administration). These data indicate that the additional modulatory effects such as apoptosis induction by miR-181a-5p adds to the therapeutic response.

Other researchers have shown that miR-181a can act as a tumor suppressor or oncogenic miRNA [36]. miR-181a-5p was found to be downregulated in non-small cell lung cancers and glioblastomas but upregulated in gastric and thyroid cancers [37,38,39,40,41]. MiR-181 family members have been reported to be downregulated in the early stages of glioma and be involved in glioma development [42]. Studies have also shown that miR-181 reduced proliferation and induced apoptosis in glioma cells [21]. Using a bioinformatic processing strategy and literature review, we identified 38 apoptosis-inducing genes that are specific to miR-181a. In various contexts, a specific miRNA could simultaneously produce competing oncogenic and tumor suppressive effects by the modulation of oncogenic or tumor suppressive mRNAs, respectively [43]. Additionally, miRNAs can modulate tumor-modifying extrinsic factors such as cancer-immune system interactions, stromal cell interactions, and sensitivity to therapy [44,45,46]. It likely the balance between these processes which determines the net outcome of whether a specific miRNA produces an oncogenic or tumor suppressive effect. In summary, although miRNA-based cancer immune therapeutics are a novel treatment strategy capable of having dual roles in both immune and tumor cells, the net response on oncogenic and tumor suppressive miRNAs must also be considered in a lineage specific manner.

## 4. Materials and Methods

### 4.1. Isolation of CD14b+ Cells from Blood and CD11b+ Cells from Glioblastoma Tissue

CD11b+ cells were isolated as described previously [7,9]. In brief, the study was approved and conducted according to protocol LAB03-0687 at The University of Texas MD Anderson Cancer Center (Houston, TX, USA). Informed consent was obtained from the patients. After the tumors had been pathologically confirmed as glioblastoma by a neuropathologist based on the World Health organization Classification of tumors of central nervous system [47], the tissue samples were collected and de-identified. The samples were washed in RPMI, broken down into small pieces, and digested with Liberase enzyme. The cells were separated by Percoll gradient. Cells were blocked for non-specific binding using FcγR-binding inhibitor (Miltenyi Biotec, Bergisch Gladbach, Germany) and CD11b magnetic beads at 4 °C for 30 min. The cell suspension was separated by a magnetic column; the negative fraction was eluted, and the positive fraction was collected and saved at −80 °C. RNA was extracted later using Trizol (Invitrogen, Carlsbad, CA, USA). CD14b+ cells (Peripheral blood mononuclear cells) were purified from matched patient blood using Ficoll gradient and the cell pellets were frozen at −80 °C. RNA was extracted using Trizol method. CD14b+ cells were isolated from healthy blood obtained as a buffy from the Gulf Coast Blood Center (Houston, TX, USA) by using Ficoll Gradient. The cells were then skewed to either M0 or M2 macrophages as described previously [8].

### 4.2. RNA Extraction and Quantitative Real-Time PCR

Total RNA from the cells (CD11b+, CD14b+, GL261, GL261 overexpressing the microRNAs or the scrambled control) was extracted using Trizol (Invitrogen) according to the manufacturer’s recommendations. RNA samples were quantified using Nanodrop (Thermofisher Scientific, Waltham, MA, USA) at OD260/280 nm. Total RNA (500 ng) was reverse transcribed into cDNA using the TaqMan MicroRNA reverse transcription kit (cat. no. 4366596, Life Technologies, Carlsbad, CA, USA) according to the manufacturer’s instructions. Quantitative reverse transcription polymerase chain reaction (RT-PCR) was performed using universal master mix (Applied Biosystems, Foster City, CA, USA) with Taqman microRNA assays (Life Technologies). For OPN, AKT, ATM, BCL2, BCL2L11, PTEN, MAP c-DNA was synthesized using the verso c-DNA synthesis kit (Thermofisher Scientific). The transcripts were analyzed by quantitative—real time PCR. The primer sequences are included in Table S2. All real-time experiments were performed on an ABI 7900 detection system (Applied Biosystems). The relative expression levels of the microRNAs were calculated using U6 internal control; Graph Pad Prism (Prism 8.0) was used to plot the curves.

### 4.3. Monocyte Culture and Differentiation

CD14b+ cells isolated from buffy were differentiated into M0 or M2 phenotypes, as described previously [8]. Briefly, CD14b+ cells were isolated from the Buffy coat fractions of human blood by density gradient centrifugation. M0 and M2 macrophages were obtained by growing the cells in RPMI+10% FBS and granulocyte macrophage colony stimulating factor (GM-CSF, 50 ng/mL) for M0 and Macrophage colony stimulating factor (M-CSF, 100 ng/mL) for M2 respectively (GMCSF & MCSF from Peprotech, Rocky Hill, NJ, USA) for 6 days. For M2 polarization other cytokines Interleukin-10 (IL-10, 10 ng/mL; Peprotech) and transforming growth factor beta-1 (TGF-β, 10 ng/mL, Peprotech) were added on Day 6 and cells were cultured for 48 h before harvest. The cytokine activity of each lot was measured by the company and was stored at appropriate conditions after receiving.

### 4.4. Quantification of OPN Using Enzyme-Linked Immunosorbent Assay

OPN levels in the supernatant were measured by enzyme-linked immunosorbent assay (ELISA) 48 h after transfection using the OPN duo set ELISA (DY1433, R&D Systems, Minneapolis, MN, USA) according to the manufacturer’s instructions.

### 4.5. Cell Culture

Murine GL261 (RRID: CVCL_Y003) and human HeLa (RRID: CVCL_0030) cell lines were purchased from the National Cancer Institute (Bethesda, MD, USA) and ATCC (Manassas, VA, USA), respectively. Cell lines from ATCC and NCI have been thoroughly tested and authenticated. The GL261 cell line was maintained in Dulbecco’s modified Eagle’s medium (Life Technologies, Carlsbad, CA, USA) supplemented with 10% FBS (GIBCO) and 1% penicillin/streptomycin at 37 °C in a humidified atmosphere of 5% CO_2_ and 95% air. HeLa cells were maintained in Eagles Essential medium supplemented with 10% FBS and 1% penicillin/streptomycin. The cells were passaged by trypsinization for 3 min at 37 °C, followed by neutralization with medium containing FBS at 1:5 dilution. All the cell lines used for this study are mycoplasma free.

### 4.6. Transient and Stable Transfections of miRNAs

miRNA mimics for miR-181a/b/c/d and scrambled control were obtained from Dharmacon. To transiently overexpress the miRNAs, we transfected M0 and M2 macrophages on day 6 with either miR-181a/b/c/d mimics or their non-targeting (NT) scrambled control using Lipofectamine 2000 (Invitrogen) according to the manufacturer’s instructions. After 48 h of transfection, cells were collected, and miR expression levels were quantified by qRT PCR. For the stable transfection of microRNAs, we purchased lentiviral vectors from Genecopeia; GL261 cells were transduced with either non-targeting control or miR-181a/b/c/d-overexpressing viruses. The cells were selected in a medium containing puromycin (2 μg/mL). The cells were expanded and analyzed for miRNA expression by quantitative PCR.

### 4.7. Luciferase Assay

To determine whether members of the miR-181 family could bind to the OPN 3′-untranslated region (UTR), we co-transfected HeLa reporter cells with the OPN 3′ UTR-dual luciferase reporter plasmid (pEZX-MT06, Genecopeia, Rockville, MD, USA) and miR-181 mimics or scrambled control (Sigma Aldrich, St. Louis, MO, USA) with Lipofectamine 2000 transfection reagent (Invitrogen). The interaction between miR-181 and its OPN were measured by comparing the results of the co-transfection of OPN 3′ UTR-luciferase reporter and miR-181 mimics with those of the 3′ UTR-luciferase reporter plasmid and the scramble control. The luciferase assay was performed using the Dual-Luciferase reporter assay system (E1910, Promega, Madison, WI, USA). Firefly luciferase activity was normalized by Renilla luciferase activity as previously described [48].

### 4.8. BrdU Proliferation Assay

Proliferation assays were performed on control and miR-overexpressing GL261 cell lines using a colorimetric BrdU assay kit (Roche, Indianapolis, IN, USA) according to the manufacturer’s recommendations. In brief, 30,000 cells per well were seeded overnight. The cells were incubated with BrdU labeling reagent for 24 h, fixed, and incubated with anti-BrdU antibody for 2 h. The substrate was added, and the color was measured at 492 nm as described previously [48].

### 4.9. Apoptosis Assay

An apoptosis assay was performed on scrambled control and miR-overexpressing GL261 cells using a Click-iT-TUNEL assay (Thermofisher Scientific). In brief, 50,000 cells were seeded in a 96-well plate overnight, the cells were fixed in 4% paraformaldehyde and permeabilized with 0.25% Triton X-100 (Sigma, Cat # X100, St. Louis, MO, USA) followed by incubation with terminal deoxynucleotidyl transferase, and the reaction mixture was incubated for 30 min. The absorbance was measured at 650 nm.

### 4.10. Caspase-3 Activity

Caspase-3 activity was measured according to the manufacturer recommendations (Catalog # K106-100, R & D systems, Minneapolis, MN, USA) using the caspase-3 calorimetric assay kit. Briefly, the cell lysates were made in ice cold RIPA buffer (Cat # 89900, Thermofisher, Waltham, MA, USA) and the lysates were quantified by the BCA protein assay (Catalog # 23227, Thermofisher, Waltham, MA, USA). The lysates were then incubated with Caspase-3 substrate, Ac-DEVD-pNA-(7-amino-4 methyl coumarin), at 37 °C for 2 h and the absorbance was measured at 405 nm. A standard curve was used to measure the caspase-3 activity and the data is represented as fold increase.

### 4.11. Syngeneic Intracranial Glioma Model

To induce intracerebral tumors in C57BL/6J mice, we collected GL261 cells or miR-overexpressing GL261 cells in logarithmic growth phase; these were washed twice with PBS and resuspended in PBS at a concentration of 5 × 10^4^ cells in 2 µL. The cells were loaded into a 25 µL Hamilton syringe with an attached 25-gauge needle. Using a Stereotactic frame (Kopf instruments, Tujunga, CA, USA), the needle was positioned 2 mm to the right of the bregma and 4 mm below the surface of the skull at the coronal suture as previously described [49] and 2 µL of cell suspension was injected. The mice were maintained in the MD Anderson Isolation Facility in accordance with Laboratory Animal Resources Commission standards and handled according to the approved protocol (00001176-RN02). The mice were randomly assigned to control and treatment groups (*n* = 6–8/group). They were observed daily and euthanized (by introduction of 100% carbon dioxide into a bedding-free cage for 2.5 min followed by cervical dislocation) when they showed signs of neurological deficit (lethargy, failure to ambulate, lack of feeding, or loss of >20% body weight). These symptoms typically occurred within 48 h prior to death. The brains were collected and fixed in 10% formalin before being embedded in paraffin.

### 4.12. In Vivo Treatments

The in vivo treatments are performed as described previously [7,12]. Briefly, the miR-181 duplex that mimics pre-miR-181 (sense: antisense) and the scrambled control miRNA duplex (sense: 5′-3′, antisense: 3′-5′) were synthesized (Boston Open Labs). The sequence of murine miR-181 is identical to that of human miR-181 on the basis of NCBI blast data [50] The treatment cohorts consisted of 20 µg of the miR-181 duplex or scrambled control in 10 µL of PBS mixed with the vehicle (80 µL of PBS containing 10 µL lipofectamine 2000; Invitrogen) or the vehicle control (90 µL PBS + 10 µL lipofectamine 2000). The mice were treated starting on day 3 after implantation, three times a week (Monday, Wednesday, and Friday) for 3 weeks.

### 4.13. Statistical Analysis

Mean and standard deviation were used to summarize the distribution of each continuous variables while frequencies and percentages were used for the distribution of each categorical variables. Continuous variables were compared between treatment groups using a two-sample *t* test. Paired groups were compared using a paired *t*-test. One-way ANOVA was used to compare multiple groups. Kaplan–Meier curves were used to estimate unadjusted time-to-event variables. The Gehan–Breslow–Wilcoxon method was used to compare each time-to-event variable between groups. *p* values less than 0.05 (two-sided) were considered statistically significant. All statistical analyses were performed using Graph Pad Prism software. All graphs represent at least three independent experiments and are done in triplicates with appropriate controls. Error bars represent SD.

### 4.14. Approval & Ethical Statement

All the human samples collection was approved and conducted according to guidelines and regulations of protocol LAB03-0687, at The University of Texas MD Anderson Cancer Center (Houston, TX, USA). The protocol was approved on 09/16/2003. Mice were maintained in the MD Anderson Isolation Facility in accordance with Laboratory Animal Resources Commission standards and handled according to Institutional Animal Care and Use Committee approved protocol (00001176-RN02) on 07/20/2015. All the experimental procedures and methods were performed in agreement with guidelines and regulations approved by the University of Texas MD Anderson Cancer Center Animal Care and Use Committee.

## 5. Conclusions

In summary, miRNA therapy or modulation is a promising strategy for developing the next generation of immune therapeutics; however, hurdles to these approaches still exist, as miRNAs may target a variety of genes, thereby triggering off-target effects. The relative ease of developing miRNA therapeutics may overcome some of the limitations that are associated with cell-based immune therapeutics. Our laboratory previously determined that three other miRNAs can be used as immune therapeutics for glioma, including miR-124, which inhibits the signal transducer and activator of transcription 3 (STAT3) [11]; miR-142-3p, which targets the transforming growth factor-β receptor [7]; and miR-138, which can inhibit immune checkpoints [12]. As such, we have been assembling a portfolio of miRNAs that can overcome immune suppression by different mechanisms and that could be administered with exosomes [51,52] or nanoparticles [14]. Given the variable expression of immune-suppressive mechanisms and pathways throughout different subtypes of glioblastoma, there may be distinct advantages to combine these for further therapeutic synergy and more comprehensive broad targeting of tumor-mediated immune suppression.

## Supplementary Materials

The following are available online at https://www.mdpi.com/2072-6694/12/12/3813/s1, Figure S1: miR-181a/b/c/d down modulated OPN in GL261 glioma cells, Figure S2: miR-181a induces apoptosis GL261 glioma cells. Caspase 3 activity was measured in the lysates from miR-181a/b/c/d overexpression clones or scrambled controls, Table S1: MicroRNAs that are predicted to bind to 3′UTR of OPN, Table S2: Primer sequences for miR-181 targets.

## References

[1] Hussain S.F., Yang D., Suki D., Aldape K., Grimm E., Heimberger A.B. (2006). The role of human glioma-infiltrating microglia/macrophages in mediating antitumor immune responses. Neuro Oncol..

[2] Prionisti I., Bühler L.H., Walker P.R., Jolivet R.B. (2019). Harnessing Microglia and Macrophages for the Treatment of Glioblastoma. Front. Pharmacol..

[3] Domingues P., González-Tablas M., Otero Á., Pascual D., Miranda D., Ruiz L., Sousa P., Ciudad J., Gonçalves J.M., Lopes M.C. (2016). Tumor infiltrating immune cells in gliomas and meningiomas. Brain Behav. Immun..

[4] Wu A., Wei J., Kong L.Y., Wang Y., Priebe W., Qiao W., Sawaya R., Heimberger A.B. (2010). Glioma cancer stem cells induce immunosuppressive macrophages/microglia. Neuro Oncol..

[5] Zhang F., Parayath N.N., Ene C.I., Stephan S.B., Koehne A.L., Coon M.E., Holland E.C., Stephan M.T. (2019). Genetic programming of macrophages to perform anti-tumor functions using targeted mRNA nanocarriers. Nat. Commun..

[6] Long K.B., Beatty G.L. (2013). Harnessing the antitumor potential of macrophages for cancer immunotherapy. Oncoimmunology.

[7] Xu S., Wei J., Wang F., Kong L.Y., Ling X.Y., Nduom E., Gabrusiewicz K., Doucette T., Yang Y., Yaghi N.K. (2014). Effect of miR-142-3p on the M2 macrophage and therapeutic efficacy against murine glioblastoma. J. Natl. Cancer Inst..

[8] Gabrusiewicz K., Rodriguez B., Wei J., Hashimoto Y., Healy L.M., Maiti S.N., Thomas G., Zhou S., Wang Q., Elakkad A. (2016). Glioblastoma-infiltrated innate immune cells resemble M0 macrophage phenotype. JCI Insight.

[9] Wei J., Marisetty A., Schrand B., Gabrusiewicz K., Hashimoto Y., Ott M., Grami Z., Kong L.Y., Ling X., Caruso H. (2019). Osteopontin mediates glioblastoma-associated macrophage infiltration and is a potential therapeutic target. J. Clin. Investig..

[10] Lund S.A., Giachelli C.M., Scatena M. (2009). The role of osteopontin in inflammatory processes. J. Cell Commun. Signal..

[11] Wei J., Wang F., Kong L.Y., Xu S., Doucette T., Ferguson S.D., Yang Y., McEnery K., Jethwa K., Gjyshi O. (2013). miR-124 inhibits STAT3 signaling to enhance T cell-mediated immune clearance of glioma. Cancer Res..

[12] Wei J., Nduom E.K., Kong L.Y., Hashimoto Y., Xu S., Gabrusiewicz K., Ling X., Huang N., Qiao W., Zhou S. (2016). miR-138 exerts anti-glioma efficacy by targeting immune checkpoints. Neuro Oncol..

[13] Xue J., Zhou A., Wu Y., Morris S.A., Lin K., Amin S., Verhaak R., Fuller G., Xie K., Heimberger A.B. (2016). miR-182-5p Induced by STAT3 Activation Promotes Glioma Tumorigenesis. Cancer Res..

[14] Yaghi N.K., Wei J., Hashimoto Y., Kong L.Y., Gabrusiewicz K., Nduom E.K., Ling X., Huang N., Zhou S., Kerrigan B.C. (2017). Immune modulatory nanoparticle therapeutics for intracerebral glioma. Neuro Oncol..

[15] Zhang M., Zhang Q., Hu Y., Xu L., Jiang Y., Zhang C., Ding L., Jiang R., Sun J., Sun H. (2017). miR-181a increases FoxO1 acetylation and promotes granulosa cell apoptosis via SIRT1 downregulation. Cell Death Dis..

[16] Chen G., Zhu W., Shi D., Lv L., Zhang C., Liu P., Hu W. (2010). MicroRNA-181a sensitizes human malignant glioma U87MG cells to radiation by targeting Bcl-2. Oncol. Rep..

[17] Ji J., Yamashita T., Budhu A., Forgues M., Jia H.L., Li C., Deng C., Wauthier E., Reid L.M., Ye Q.H. (2009). Identification of microRNA-181 by genome-wide screening as a critical player in EpCAM-positive hepatic cancer stem cells. Hepatology.

[18] Li N., Cheng C., Wang T. (2020). miR-181c-5p Mitigates Tumorigenesis in Cervical Squamous Cell Carcinoma via Targeting Glycogen Synthase Kinase 3β Interaction Protein (GSKIP). OncoTargets Ther..

[19] Li Y., Kuscu C., Banach A., Zhang Q., Pulkoski-Gross A., Kim D., Liu J., Roth E., Li E., Shroyer K.R. (2015). miR-181a-5p Inhibits Cancer Cell Migration and Angiogenesis via Downregulation of Matrix Metalloproteinase-14. Cancer Res..

[20] Wang H., Tao T., Yan W., Feng Y., Wang Y., Cai J., You Y., Jiang T., Jiang C. (2015). Upregulation of miR-181s reverses mesenchymal transition by targeting KPNA4 in glioblastoma. Sci. Rep..

[21] Zhai F., Chen X., He Q., Zhang H., Hu Y., Wang D., Liu S., Zhang Y. (2019). MicroRNA-181 inhibits glioblastoma cell growth by directly targeting CCL8. Oncol. Lett..

[22] Wen X., Li S., Guo M., Liao H., Chen Y., Kuang X., Liao X., Ma L., Li Q. (2020). miR-181a-5p inhibits the proliferation and invasion of drug-resistant glioblastoma cells by targeting F-box protein 11 expression. Oncol. Lett..

[23] Adams J.M., Cory S. (2007). The Bcl-2 apoptotic switch in cancer development and therapy. Oncogene.

[24] Willis S., Day C.L., Hinds M.G., Huang D.C. (2003). The Bcl-2-regulated apoptotic pathway. J. Cell Sci..

[25] Yip K.W., Reed J.C. (2008). Bcl-2 family proteins and cancer. Oncogene.

[26] Lessene G., Czabotar P.E., Colman P.M. (2008). BCL-2 family antagonists for cancer therapy. Nat. Rev. Drug Discov..

[27] Zhu T., Shen Y., Tang Q., Chen L., Gao H., Zhu J. (2014). BCNU/PLGA microspheres: A promising strategy for the treatment of gliomas in mice. Chin. J. Cancer Res..

[28] Wu X.F., Zhou Z.H., Zou J. (2017). MicroRNA-181 inhibits proliferation and promotes apoptosis of chondrocytes in osteoarthritis by targeting PTEN. Biochem. Cell Biol..

[29] Xu C.H., Xiao L.M., Zeng E.M., Chen L.K., Zheng S.Y., Li D.H., Liu Y. (2019). MicroRNA-181 inhibits the proliferation, drug sensitivity and invasion of human glioma cells by targeting Selenoprotein K (SELK). Am. J. Transl. Res..

[30] Zhang X., Nie Y., Li X., Wu G., Huang Q., Cao J., Du Y., Li J., Deng R., Huang D. (2014). MicroRNA-181a functions as an oncomir in gastric cancer by targeting the tumour suppressor gene ATM. Pathol. Oncol. Res..

[31] Wang Q., Hu B., Hu X., Kim H., Squatrito M., Scarpace L., de Carvalho A.C., Lyu S., Li P., Li Y. (2017). Tumor Evolution of Glioma-Intrinsic Gene Expression Subtypes Associates with Immunological Changes in the Microenvironment. Cancer Cell.

[32] Chen P., Zhao D., Li J., Liang X., Li J., Chang A., Henry V.K., Lan Z., Spring D.J., Rao G. (2019). Symbiotic Macrophage-Glioma Cell Interactions Reveal Synthetic Lethality in PTEN-Null Glioma. Cancer Cell.

[33] Gimba E.R.P., Brum M.C.M., De Moraes G.N. (2019). Full-length osteopontin and its splice variants as modulators of chemoresistance and radioresistance (Review). Int. J. Oncol..

[34] Han B., Huang J., Han Y., Hao J., Wu X., Song H., Chen X., Shen Q., Dong X., Pang H. (2019). The microRNA miR-181c enhances chemosensitivity and reduces chemoresistance in breast cancer cells via down-regulating osteopontin. Int. J. Biol. Macromol..

[35] Icer M.A., Gezmen-Karadag M. (2018). The multiple functions and mechanisms of osteopontin. Clin. Biochem..

[36] Semonche A., Shah A.H., Ivan M.E., Komotar R.J. (2019). Towards a microRNA-based Gene Therapy for Glioblastoma. Neurosurgery.

[37] Gao W., Yu Y., Cao H., Shen H., Li X., Pan S., Shu Y. (2010). Deregulated expression of miR-21, miR-143 and miR-181a in non small cell lung cancer is related to clinicopathologic characteristics or patient prognosis. Biomed. Pharmacother..

[38] Shi L., Cheng Z., Zhang J., Li R., Zhao P., Fu Z., You Y. (2008). hsa-mir-181a and hsa-mir-181b function as tumor suppressors in human glioma cells. Brain Res..

[39] Chen G., Shen Z.L., Wang L., Lv C.Y., Huang X.E., Zhou R.P. (2013). Hsa-miR-181a-5p expression and effects on cell proliferation in gastric cancer. Asian Pac. J. Cancer Prev..

[40] Keutgen X.M., Filicori F., Crowley M.J., Wang Y., Scognamiglio T., Hoda R., Buitrago D., Cooper D., Zeiger M.A., Zarnegar R. (2012). A panel of four miRNAs accurately differentiates malignant from benign indeterminate thyroid lesions on fine needle aspiration. Clin. Cancer Res..

[41] Zhang X., Nie Y., Du Y., Cao J., Shen B., Li Y. (2012). MicroRNA-181a promotes gastric cancer by negatively regulating tumor suppressor KLF6. Tumour Biol..

[42] Yang L., Ma Y., Xin Y., Han R., Li R., Hao X. (2018). Role of the microRNA 181 family in glioma development. Mol. Med. Rep..

[43] Svoronos A.A., Engelman D.M., Slack F.J. (2016). OncomiR or Tumor Suppressor? The Duplicity of MicroRNAs in Cancer. Cancer Res..

[44] Omar H.A., El-Serafi A.T., Hersi F., Arafa E.A., Zaher D.M., Madkour M., Arab H.H., Tolba M.F. (2019). Immunomodulatory MicroRNAs in cancer: Targeting immune checkpoints and the tumor microenvironment. FEBS J..

[45] Orso F., Quirico L., Dettori D., Coppo R., Virga F., Ferreira L.C., Paoletti C., Baruffaldi D., Penna E., Taverna D. (2020). Role of miRNAs in tumor and endothelial cell interactions during tumor progression. Semin. Cancer Biol..

[46] He B., Zhao Z., Cai Q., Zhang Y., Zhang P., Shi S., Xie H., Peng X., Yin W., Tao Y. (2020). miRNA-based biomarkers, therapies, and resistance in Cancer. Int. J. Biol. Sci..

[47] Louis D.N., Ohgaki H., Wiestler O.D., Cavenee W.K., Burger P.C., Jouvet A., Scheithauer B.W., Kleihues P. (2007). The 2007 WHO classification of tumours of the central nervous system. Acta Neuropathol..

[48] Marisetty A.L., Singh S.K., Nguyen T.N., Coarfa C., Liu B., Majumder S. (2017). REST represses miR-124 and miR-203 to regulate distinct oncogenic properties of glioblastoma stem cells. Neuro Oncol..

[49] Heimberger A.B., Crotty L.E., Archer G.E., Hess K.R., Wikstrand C.J., Friedman A.H., Friedman H.S., Bigner D.D., Sampson J.H. (2003). Epidermal growth factor receptor VIII peptide vaccination is efficacious against established intracerebral tumors. Clin. Cancer Res..

[50] National Center for Biotechnology Information, BLAST. https://blast.ncbi.nlm.nih.gov/Blast.cgi.

[51] Graner M.W., Schnell S., Olin M.R. (2018). Tumor-derived exosomes, microRNAs, and cancer immune suppression. Semin. Immunopathol..

[52] Barros F.M., Carneiro F., Machado J.C., Melo S.A. (2018). Exosomes and Immune Response in Cancer: Friends or Foes?. Front. Immunol..

