# Supplementary material for: MiR-181 Family Modulates Osteopontin in Glioblastoma Multiforme
Source: Cancers (Basel). 2020 Dec 17;12(12):3813. doi: 10.3390/cancers12123813 (PMC7765845; doi:10.3390/cancers12123813)
Supplement: Supplementary file 1 [file cancers-12-03813-s001.pdf]

Supplementary Materials: MiR-181 Family Modulates Osteopontin in Glioblastoma Multiforme

Anantha Marisetty, Jun Wei, Ling-Yuan Kong, Martina Ott, Dexing Fang, Aria Sabbagh and Amy B. Heimberger

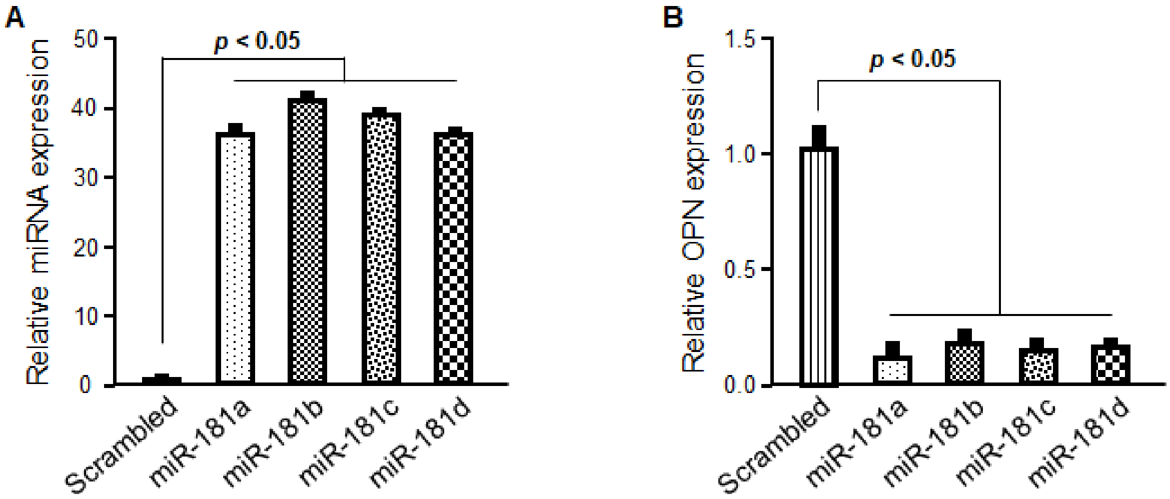

Figure S1. miR-181a/b/c/d down modulated OPN in GL261 glioma cells. (**A**) GL261 cells were stably transfected to overexpress either non-targeting control or miR-181a/b/c/d-5p. Overexpression of miR-181a/b/c/d was confirmed by qRT-PCR. (**B**) qRT-PCR analysis of OPN expression in GL261 cells overexpressing miR-181 mimics. Knockdown of OPN was observed upon overexpression of miR-181a/b/c/d.

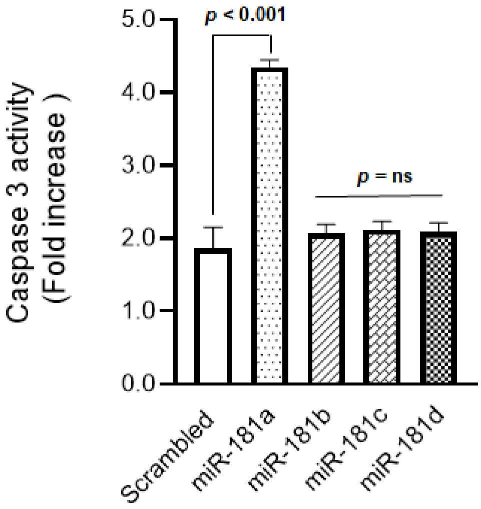

Figure S2. miR-181a induces apoptosis GL261 glioma cells. Caspase 3 activity was measured in the lysates from miR-181a/b/c/d overexpression clones or scrambled controls. Data represented is fold increase in caspase -3 activity ($n = 3$). Caspase -3 activity is significant upon miR-181a overexpression while with miR-181b/c/d is not significant.

Table S1. MicroRNAs that are predicted to bind to 3′UTR of OPN.

<table>
<tr><th>Conserved microRNAs</th><th>Predicted Consequential Pairing of Target Region (top) and miRNA (Bottom)</th><th>Site Type</th><th>Context ++ Score</th></tr>
<tr><td>Position 105-111 of OPN 3′UTR hsa-miR-181a-5p</td><td>5′• • • UCUCAGUUUAUUGGUUGAAUGUG • • •
| | | | | | | | | | | | |
3′• • • UGAGUGGCUGUCGCAACUUACAA • • •</td><td>7mer - 8mer</td><td>−0.21</td></tr>
<tr><td>Position 105-111 of OPN 3′UTR hsa-miR-181b-5p</td><td>5′• • • UCUCAGUUUAUUGGUUGAAUGUG • • •
| | | | | | | | | |
3′• • • UGGGUGGCUGUCGUUACUUACAA • • •</td><td>7mer - 8mer</td><td>−0.19</td></tr>
<tr><td>Position 105-111 of OPN 3′UTR hsa-miR-181c-5p</td><td>5′• • • UCUCAGUUUAUUGGUUGAAUGUG • • •
| | | | | | | | | | |
3′• • • UGAGUGGCUGUCCAACUUACAA • • •</td><td>7mer - 8mer</td><td>−0.21</td></tr>
<tr><td>Position 105-111 of OPN 3′UTR hsa-miR-181d-5p</td><td>5′• • • UCUCAGUUUAUUGGUUGAAUGUG • • •
| | | | | | | | | |
3′• • • UGGGUGGCUGUUGUUACUUACAA • • •</td><td>7mer - 8mer</td><td>−0.19</td></tr>
<tr><td>Position 105-111 of OPN 3′UTR hsa-miR-4262</td><td>5′• • • UCUCAGUUUAUUGGUUGAAUGUG • • •
| | | | | | | | |
3′• • • GUCCAUCAGACUUACAG • • •</td><td>7mer - 8mer</td><td>−0.16</td></tr>
</table>

Table S2. Primer sequences for miR-181 targets.

Gene	Forward Sequence	Reverse Sequence
AKT	5′-TCTATGGCGCTG AGATTGTG-3′	5′-CTTAATGTGCCCGTC CTTGT-3′
ATM	5′- ATCCCTTGTGTGTTCTCTG −3′	5′- CGCCTCTGCTGTCGTGTA T −3′
BCL2	5′-CCTGTGGATGACTGAGTACC-3′	5′-GAGACAGCCAGGAGAAATCA-3′
BCL2L11	5′-ATGTCTGACTCTGACTCTCG-3′	5′-CCTTGTGGCTCTGTCTGTAG-3′
PTEN	5′-CCGAAAGGTTTGCTACCATTCT-3′	5′-AAAATTATTTCCTTTCTGAGCATTCC-3′
MAPK	5′-AGGGCGATGTGACGTTT-3′	5′-CTGGCAGGGTGAAGTTGG-3′
OPN	5′-TCTGATGAGACCGTCACTGC-3′	5′-TCTCCTGGCTCTCTTTGGAA-3′
GAPDH	5'-TGCATCCTGCACCACCAA CT-3'	5'-TGCCTGCTTCACCACCTTC-3'

Publisher's Note: MDPI stays neutral with regard to jurisdictional claims in published maps and institutional affiliations.

© 2020 by the authors. Licensee MDPI, Basel, Switzerland. This article is an open access article distributed under the terms and conditions of the Creative Commons Attribution (CC BY) license (http://creativecommons.org/licenses/by/4.0/).
